# On the Amount of Utility of Permanent Exutories in the Treatment of Chronic Diseases

**Published:** 1859-09

**Authors:** M. Zuskowski


*On the amount of utility of Permanent Exutories in the treatment of Chronic Diseases. By M. Zuskowski.*

1st. *Permanent Exutories in Chronic Phlegmasia.*—Lesions which result from chronic inflammation, when exempt from all diathesic influence, are generally, even after a very long period, susceptible of resolution. It is especially in disease of the articulations that the greatest number of successful cases have been observed. Of 58 of the cases of Pott's disease and white swellings, the exutories were the sole means employed in 22; they were used in conjunction with other means in 12, and in 24 they were resorted to after other measures had failed. A no less positive amount of success has attended their use in chronic myelitis and the consequent paralysis—44 instances of recovery from such paralysis, with or without vertebral disease, being on record. Of 20 cases of amaurosis, 7 were treated exclusively by exutories, and 13 after the failure of other means; permanent success resulting in the whole. So with 30 cases of various descriptions of ophthalmía, the great bulk of which had previously been treated without success. Besides these may be mentioned old cases of pleuritic and peritoneal effusion.

2. *Exutories in Tuberculization.*— The author reports ten instances of pulmonary consumption treated with success by exutories. These individuals were all the issue of healthy parents, with no antecedent phthisis in their families. There were no concomitant nor anterior abdominal affections, signs of scrofula, or disease of the bones or joints, but all the patients had cavities at the upper part of the lung, accompanied by the usual cortege of symptoms.

3. *Exutories in Neuroses.*—Their beneficial effect has been observed in ten various forms of these, whether relating to modifications of sensibility, motility or impressionability, or to aberrations of the perceptions of the intellectual powers, or of the moral and affective faculties. Seeing, then, how useful this means may often prove, how comes it that it has fallen into discredit? By reason of the abuse which arose from its indiscriminate employment, whether suitable indications were present or not. Among the conditions which should oppose the use of permanent exutories as a means of treating chronic disease, are the following:

1st. *Deep seated alteration of structure.*—For example, the atrophy or melting down of an organ, which has already given rise to symptoms of resorption or colliquation. In subjects placed even in the most favorable conditions, if the organs have undergone deep-seated alterations, if the general reaction is continuous, giving rise to disturbance of some important function, and especially if nutrition be already deeply impaired, not only have exutories no longer any chance of success, but they may even hasten the fatal termination.

2d. *Degenerations.*—Without speaking here of primary heteromorphies, for which no one would think of applying exutories, we allude to those insidious transformations of simply indurated or hypertrophied tissues, which are brought about either by the sole effect of chronicity, or under the influence of some diathesic or hereditary condition.

3d. *Tuberculization.*—Although exutories may exert a beneficial action in cases of isolated tubercles, limited to a circumscribed portion of an organ, they offer no chance of success in general tuberculization—that is, when the diseased process has been set up in several organs at once, or even in several parts of the same organ.

4th. *Hereditary influence.*—This exerts great pathogenic influence in chronic disease. Next to tubercular affections, it is in the neuroses especially that it plays so immense a part. In the examples of epilepsy and insanity, in which exutories have proved useful, the patients have been exempt from this fatal influence. Unfortunately, these are the rarest cases; the immense majority are subjected to hereditary influence, and exutories will fail to exert any salutary effect on them.—*Medico-Chir. Rev.*

